# Characterization of Bonding Defects in Fiber-Reinforced Polymer-Bonded Structures Based on Ultrasonic Transmission Coefficient

**DOI:** 10.3390/ma17051080

**Published:** 2024-02-27

**Authors:** Zeqi Bian, Bin Wu, Bing Liu, Yan Lyu, Jie Gao, Cunfu He

**Affiliations:** 1 College of Mechanical & Energy Engineering, Beijing University of Technology, Beijing 100124, China; bianzq@emails.bjut.edu.cn (Z.B.);; 2 Faculty of Information Technology, Beijing University of Technology, Beijing 100124, China

**Keywords:** fiber-reinforced polymer, bonded structures, ultrasonic transmission coefficient, interfacial slip, debonding defects

## Abstract

This research delves into the characterization of the ultrasonic transmission coefficient pertaining to various types of bonding defects in Fiber-Reinforced Polymer (FRP)-bonded structures. Initially, an ultrasonic transmission coefficient calculation model for FRP-bonded structures in a water immersion environment is established. This model is used to analyze the variation in the ultrasonic transmission coefficient under different defect types, namely intact bonding, interfacial slip, and debonding defects. Subsequently, a frequency domain finite element analysis model of FRP-bonded structures with different defect types is constructed. The simulation validates the accuracy of the theoretical analysis results and concurrently analyzes the variation in the transmission signal when the defects alter. Lastly, an experimental platform for water immersion ultrasonic transmission measurement is set up. The transmission signals under different defect types are extracted through experiments and evaluated in conjunction with theoretical calculations to assess the types of bonding defects.

## 1. Introduction

Fiber-Reinforced Polymers (FRPs), celebrated for their exceptional strength-to-weight ratio, fatigue endurance, and resistance to corrosion, are ideally suited for integration into bonded structures crafted from carbon fiber or glass fiber composite materials. These structures are extensively employed in the fabrication of lightweight constructs, with applications spanning aerospace, wind turbine blades, and contemporary automobiles [1,2]. Nevertheless, the bonded segment of the FRP structure is vulnerable to environmental influences and process-related variables. This vulnerability frequently results in slippage and debonding at the bonding interface, thereby undermining the structural integrity of the bonded assembly.

The necessity for non-destructive testing and evaluation of the bonding quality in bonded structures is unequivocal. Dolati et al. [3] provide an in-depth analysis of the state-of-the-art nondestructive characterization techniques currently employed for assessing the bond quality of structures bonded with Fiber-Reinforced Polymers (FRPs). Each nondestructive testing method is meticulously examined, with detailed discussions on the theoretical underpinnings, methodologies, advantages, and limitations. Among these methods, Infrared Thermography (IR), Ground Penetrating Radar (GPR), and Ultrasonic Testing (UT) are identified as the most effective techniques for inspecting FRP-bonded structures. In this context, non-destructive testing methodologies predicated on ultrasonic performance parameters have gained significant traction [4]. This domain has been the focus of numerous studies. For instance, Piao et al. [5] conducted measurements of the adhesive interface in thermoplastic composites. They utilized an ultrasonic phased array in conjunction with an intelligent algorithm, demonstrating a novel methodology in this field of study. Park et al. [6] leveraged a laser ultrasonic scanning platform for the automated and visual detection of debonding defects in CFRP airfoils and delamination defects in GFRP fan blades. Sasmal et al. [7] implemented an innovative approach to detect bond defects in Fiber-Reinforced Polymer (FRP)—concrete-bonded structures. They employed a variety of linear and nonlinear ultrasonic methods, which proved to be effective in detecting dimensional changes in bond defects. Billson et al. [8] utilized the ultrasonic pulse echo method to identify poorly bonded areas on the bonding interface via computer modeling analysis. Pan Q et al. [9] explored the correlation between bonding defects and ultrasonic echo amplitude, propagation time, and phase in resin carbon fiber and aluminum bonding parts, validating the accuracy and reliability of this relationship through the test results of multilayer specimens containing defects. Sarr et al. [10] performed experimental measurements on voids, debonding, and weak bonding defects in bonded structures using ultrasonic testing, employing a random forest algorithm to differentiate defect types. Numerous methods have been developed and have significantly advanced the field of defect detection in bonded structures. However, the current focus of these techniques is predominantly on the identification of debonding defects. It is important to note that adhesive interface slip is another critical factor leading to the failure of adhesive structures. Despite its significance, there is a lack of methods reported in the literature that can effectively detect and identify slip defects.

Conversely, recent studies have demonstrated that ultrasonic reflection and transmission coefficients are sensitive to the state of the adhesive interface in bonded structures. This sensitivity presents a potential avenue for not only detecting but also differentiating between various forms of failure within the bonded structure. Most of the current research focuses on theoretical aspects. For instance, Michaloudaki et al. [11] examined the ultrasonic bulk wave propagation characteristics under conditions of rigid connection and debonding at the interface, considering the matrix of the aluminum/epoxy/aluminum three-layer bonding structure as a semi-infinite solid space. Tattersall et al. [12] derived expressions for the ultrasonic bulk wave reflection and transmission coefficients for the solid/solid bonding interface. Mori et al. [13] deduced the ultrasonic reflection/transmission coefficient expression for the bonding structure of isotropic materials under water immersion conditions, introducing a spring model at the boundary of the bonding interface and investigating the variation of the corresponding ultrasonic reflection/transmission coefficient spectral curves when the single-bond interface weakens and when the double-bond interface weakens in the bonding structure. He et al. [14] established the ultrasonic theoretical analysis and finite element simulation model of the bonding structure composites based on the matrix method and the spring model method, studying the impact of the weakening of the bonding interface and the weakening of the cohesion of the adhesive layer on the reflection/transmission coefficient. And in terms of reflection/transmission coefficients detection, Li et al. [15] conducted an experimental investigation into the ultrasonic characterization of bond strength degradation. They quantified the degree of bond strength weakening by tracking the shift in the extreme point of the ultrasonic reflection transmission coefficient. These two studies offered both theoretical and experimental insights into the ultrasonic nondestructive characterization method for assessing the degree of adhesive strength weakening in bonded structures. However, their focus was primarily on characterizing the weakening of bond strength for undebonded interfaces. There remains a gap in the literature regarding effective theoretical and experimental guidance when debonding or slip defects occur in bonded structures. In conclusion, the field of non-destructive testing of bonded structures, particularly those involving FRP, is a vibrant and rapidly progressing research area. The aforementioned studies offered valuable insights into the challenges and potential solutions related to ensuring the integrity of these structures. However, additional research is warranted to enhance the accuracy and reliability of these testing methodologies.

Current research on the acoustic detection of interface defects in bonded structures is primarily focused on isotropic materials. Despite their intricate acoustic wave propagation characteristics, anisotropic composite materials have been relatively overlooked. This research gap highlights the necessity for more extensive studies on anisotropic composite materials. When acoustic waves propagate and encounter an interface, they interact with it in a complex manner. The characteristics of the interface significantly affect wave propagation characteristics. Hence, it is crucial to study the impact of interface defects on ultrasonic wave propagation characteristics. In conclusion, there is an urgent need for more experimental research and studies on anisotropic composite materials in the field of acoustic detection of interface defects in bonded structures. Such research can not only address a critical gap in the existing literature, but also contribute to the development of more precise and dependable non-destructive testing methods. Indeed, the detection methods for interfacial slip defects in bonded structures are scarcely reported in the literature. This represents a significant category of adhesive interface failures, characterized by tangential connectivity but normal freedom at the adhesive interface. The development of a nondestructive testing method capable of differentiating between intact bonding, weak bonding, interfacial slip, and complete debonding is of paramount importance for ensuring the safety of engineering structures.

In this study, we propose a non-destructive testing method for identifying the failure modes of adhesive interfaces, utilizing the ultrasonic transmission coefficient as the primary means of detection. The viability of this detection method is thoroughly examined through theoretical calculations, simulation analyses, and experimental studies. First, based on the global matrix method, the ultrasonic reflection/transmission coefficient expressions of the FRP bonded structures with slip and debonding interfaces under liquid immersion conditions are deduced. Second, the time/frequency domain simulation study of the ultrasonic transmission characteristics of the FRP bonding structure is carried out, which verifies the correctness of the theoretical derivation results and explores the acquisition method of the transmission characteristics in the time/frequency domain. Finally, the type of debonding for different bonding results is measured by a combination of theoretical and experimental measurements. The detection method proposed in this paper can effectively detect and differentiate between debonding and slipping defects at the bonding interface.

## 2. Theoretical Analysis

**Figure 1:**
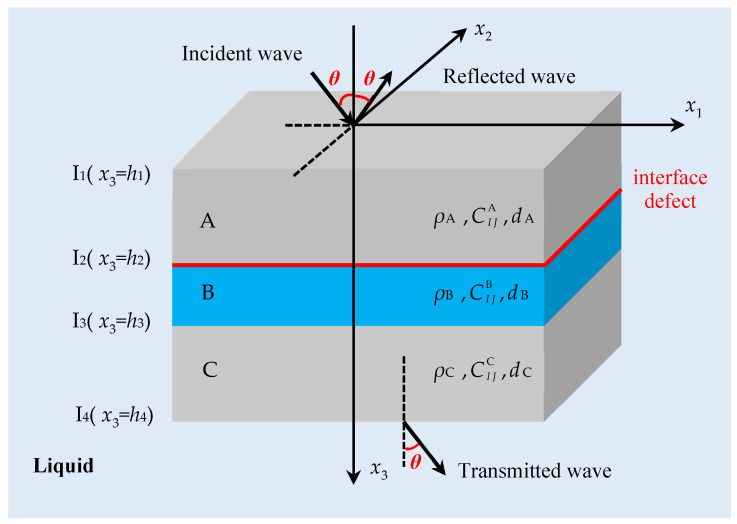
Theoretical modeling of FRP-bonded structures.

The theoretical analysis model of the FRP bonding structure with an interface defect is shown in Figure 1. The obliquely incident plane longitudinal wave is incident on the structure along the direction of the angle *θ* with the *x*_3_axis, and the reflection and transmission phenomena appear on the upper and lower surfaces of the bonded structure, respectively. According to Snell’s law, the propagation direction vectors of the incident wave, the reflected wave and the transmitted wave are in the *x*_1_ − *x*_3_ plane, and the propagation angle is equal to *θ*. Among them, *d*_A_ (m), *d*_B_ (m), *d*_C_ (m) are the thicknesses of substrate layer A, adhesive layer B and substrate layer C, respectively, *ρ*_1_ (kg/m^3^) and $C_{IJ}^{1}$ (Pa), *ρ*_2_ (kg/m^3^) and $C_{IJ}^{3}$ (Pa), *ρ*_3_ (kg/m^3^)and $C_{IJ}^{3}$ (Pa), (I,J = 1,2,…,6) are the density and elastic constant of substrate layer A, adhesive layer B and substrate layer C, respectively. It should be noted that the fluids discussed in this paper are assumed to be ideal fluids, and the viscous effect is not considered.

For linear elastic materials, under small deformation and no physical force, the constitutive equation of the FRP layer or the adhesive layer can be expressed as follows:

(1) $$\sigma_{ij}=C_{ijkl}\varepsilon_{kl},(i,j,k,l=1,2,3)$$

Displacement and strain equations are

(2) $$\varepsilon_{ij}=\frac{1}{2}\left(\frac{\partial u_{i}}{\partial x_{j}}+\frac{\partial u_{j}}{\partial x_{i}}\right)\;,\left(i,j=1,2,3\right)$$

Equation of motion is

(3) $$\frac{\partial \sigma_{ij}}{\partial x_{j}}=\rho \frac{\partial^{2}u_{i}}{\partial t^{2}},(i,j=1,2,3)$$

where *u_i_* (m), *ε_ij_*, *σ_ij_* (Pa) are displacement, strain and stress components, respectively. *C_ijkl_* (Pa) is the elastic constant and *ρ* (kg/m^3^) is material density.

Assuming that the propagation direction vector of the sound wave in the solid is $\vec{p}=\left(1,0,\alpha \right)$, the displacement of the sound field propagating in the solid can be expressed as

(4) $$\begin{array}{l} u_{1}=U_{1}\cdot e^{jk(x_{1}+\alpha x_{3}-ct)}\\ u_{2}=U_{2}\cdot e^{jk(x_{1}+\alpha x_{3}-ct)}\\ u_{3}=U_{3}\cdot e^{jk(x_{1}+\alpha x_{3}-ct)} \end{array}$$

where *U*_1_, *U*_2_, and *U*_3_ are the displacement amplitudes in the *x*_1_, *x*_2_, and *x*_3_ directions, respectively, and *k* (m^−1^) and *c* (m/s) are the wavenumber and phase velocity in the *x*_1_ direction, respectively. *α* is the ratio of the wave number in the *x*_3_ axis direction to the wave number in the *x*_1_ axis direction in the medium. We substitute Equations (1), (2) and (4) into Equation (3) to obtain the displacement and stress expressions in the solid layer:

(5) $${\left\{u_{1}^{n},u_{2}^{n},u_{3}^{n},\sigma_{33}^{n},\sigma_{13}^{n},\sigma_{23}^{n}\right\}}^{T}={\left[R^{n}\left(x_{3}\right)\right]}_{6\times 6}\cdot {\left\{N_{1}^{n},N_{2}^{n},N_{3}^{n},N_{4}^{n},N_{5}^{n},N_{6}^{n}\right\}}^{T}$$

In the above formula, *n* = A, B, C correspond to FRP layer A, adhesive layer B and FRP layer C, respectively. [*R*(*x*_3_)] is the property matrix of the solid layer, and its elements are related to the material properties and wavenumbers, and the matrix elements are shown in Appendix A. $N_{1}^{n}$, $N_{2}^{n}$, $N_{3}^{n}$, $N_{4}^{n}$, $N_{5}^{n}$, $N_{6}^{n}$ are the expansion coefficients of the local wave.

When *x*_3_ ≤ 0 and *x*_3_ ≥ *h*_4_, the displacement component in the liquid can be expressed as:

(6) $$\left\{ \begin{array}{l} u_{1}^{0}=A_{0}\cdot 1\cdot e^{jk(x_{1}+\varphi x_{3}-ct)}+A_{R}\cdot 1\cdot e^{jk(x_{1}-\varphi x_{3}-ct)}\\ u_{3}^{0}=A_{0}\cdot \varphi \cdot e^{jk(x_{1}+\varphi x_{3}-ct)}-A_{R}\cdot \varphi \cdot e^{jk(x_{1}-\varphi x_{3}-ct)} \end{array}\right.\begin{array}{cc} & x_{3}\leq 0 \end{array}$$

(7) $$\left\{ \begin{array}{l} u_{1}^{h_{4}}=A_{T}\cdot 1\cdot e^{jk(x_{1}+\varphi x_{3}-ct)}\\ u_{3}^{h_{4}}=A_{T}\cdot \varphi \cdot e^{jk(x_{1}+\varphi x_{3}-ct)} \end{array}\right.\begin{array}{cc} & x_{3}\geq h_{4} \end{array}$$

where *φ* = cot*θ*, *A*_0_, *A*_R_ and *A*_T_ are the displacement amplitudes of the incident, reflected and transmitted waves, respectively. Therefore, reflection coefficient *R* = *A*_R_/*A*_0_, and transmission coefficient *T* = *A*_T_/*A*_0_. The stress in the liquid is [16,17]

(8) $$\left\{ \begin{array}{l} \sigma =K_{w}(\nabla \cdot u_{w})\\ K_{w}=\rho_{w}c_{w}^{2}\\ u_{w}=\left(u_{1},u_{2},u_{3}\right) \end{array}\right.$$

where *K*_w_ is the compressibility of the liquid, *c*_w_ (m/s) is the speed of sound of the liquid, and *ρ*_w_ (kg/m^3^) is the density of the liquid. When *x*_3_ ≤ 0 and *x*_3_ ≥ *h*_4_, the stress in the liquid is

(9) $$\left\{ \begin{array}{l} \sigma_{33}^{0}=K_{w}\left(1+\varphi^{2}\right)\left(jkA_{0}\cdot e^{jk(x_{1}+\varphi x_{3}-ct)}+jkA_{R}\cdot e^{jk(x_{1}-\varphi x_{3}-ct)}\right)\\ \sigma_{13}^{0}=0 \end{array}\right.\begin{array}{cc} & x_{3}\leq 0 \end{array}$$

(10) $$\left\{ \begin{array}{l} \sigma_{33}^{h_{4}}=K_{w}\left(1+\varphi^{2}\right)\cdot jkA_{T}\cdot e^{jk(x_{1}+\varphi x_{3}-ct)}\\ \sigma_{13}^{h_{4}}=0 \end{array}\right.\begin{array}{cc} & x_{3}\geq h_{4} \end{array}$$

At the liquid–solid interface, the normal displacement and stress are continuous, so the interface conditions of the interfaces I_1_ and I_4_ are

(11) $${u_{3}^{A}=u_{3}^{0},\sigma_{33}^{A}=\sigma_{33}^{0},\sigma_{13}^{A}=\sigma_{13}^{0}=0,\sigma_{23}^{A}=\sigma_{23}^{0}=0|}_{x_{3}=0}$$

(12) $${u_{3}^{C}=u_{3}^{h_{4}},\sigma_{33}^{C}=\sigma_{33}^{h_{4}},\sigma_{33}^{C}=\sigma_{13}^{h_{4}}=0,\sigma_{23}^{C}=\sigma_{23}^{h_{4}}=0|}_{x_{3}=h_{4}}$$

When the upper interface slips and the lower interface is well bonded, interface I_2_ is the slip interface. Normal displacement *u*_3_ and stress *σ*_33_ at the interface are continuous, tangential stresses *σ*_13_ and *σ*_23_ are zero, and tangential displacements *u*_1_ and *u*_2_ are discontinuous. The interface conditions can be expressed as

(13) $${u_{3}^{A}=u_{3}^{B},\;\sigma_{33}^{A}=\sigma_{33}^{B},\sigma_{13}^{A}=\sigma_{13}^{B}=0,\;\sigma_{23}^{A}=\sigma_{23}^{B}=0|}_{x_{3}=h_{2}}$$

Interface I_3_ is a rigid connection interface, the normal displacement and stress are continuous, and the tangential displacement and stress are also continuous. The interface conditions can be expressed as

(14) $${u_{1}^{B}=u_{1}^{C},u_{2}^{B}=u_{2}^{C},u_{3}^{B}=u_{3}^{C},\sigma_{33}^{B}=\sigma_{33}^{C},\sigma_{13}^{B}=\sigma_{13}^{C},\sigma_{23}^{B}=\sigma_{23}^{C}|}_{x_{3}=h_{3}}$$

The global matrix method obtains a single matrix equation by satisfying the boundary conditions on all interfaces at the same time, and the corresponding solution can offer the properties of waves in all layers, which can effectively solve the problem of numerical instability. Therefore, this paper adopts the global matrix modeling technique.

Simultaneously combining Equations (11)~(14), the global matrix equation containing the reflection/transmission coefficients is obtained as shown in Equation (15). Among them, block matrices [*G*(0)], [*H* (*h*_4_)], [*L*^A^(*h*_2_)] and [*K*^B^(*h*_2_)] are shown in Appendix B.

(15) $$\begin{array}{l} {\left[\begin{array}{cccc} \left[Q^{A}\left(0\right)\right] & & & \left[G(0)\right]\\ \left[L^{A}\left(h_{2}\right)\right] & -\left[K^{B}\left(h_{2}\right)\right] & & \\ & -\left[R_{m}^{B}(h_{3})\right] & \left[R_{m}^{C}(h_{3})\right] & \\ & & \left[Q^{C}\left(h_{4}\right)\right] & \left[H\left(h_{4}\right)\right] \end{array}\right]}_{\;20\times 20}\cdot {\left[\begin{array}{c} \left\{N_{i}^{A}/A_{0}\right\}\\ \left\{N_{i}^{B}/A_{0}\right\}\\ \left\{N_{i}^{C}/A_{0}\right\}\\ \left\{\begin{array}{l} A_{R}/A_{0}\\ A_{T}/A_{0} \end{array}\right\} \end{array}\right]}_{\;20\times 1}\\ ={\left[\begin{array}{c} \varphi \\ K_{w}jk\left(1+\varphi^{2}\right)\\ {\left\{0\right\}}_{18\times 1} \end{array}\right]}_{\;20\times 1}\;,\;(i=1,2,3,4,5,6)\; \end{array}$$

Matrices [*Q*^A^(0)] and [*Q*^C^(*h*_4_)] are 4 × 6 matrices corresponding to substrate layer A and substrate layer C, respectively, and the matrix elements are taken from the third to sixth rows of material property matrix [*R*(*x*_3_)] in Appendix A.

**Figure 2:**
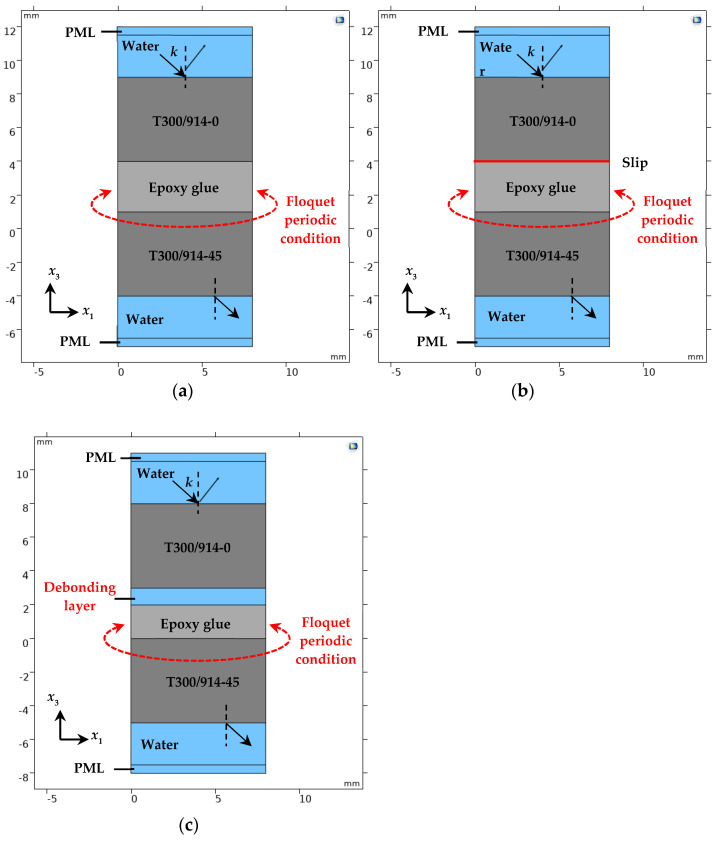
Frequency domain finite element modeling of FRP−bonded structures. (**a**) Perfect bonding; (**b**) Interface slip; (**c**) Interface debonding.

The theoretical analysis model of the FRP bonding structure with the debonding interface is shown in Figure 2. For the bonding structure of anisotropic materials, due to the debonding of the interface, the fluid enters the debonding gap to form a certain fluid layer. At this time, the debonding interface is the fluid–solid interface and the bonding structure becomes the “four-layer structure”.

For the FRP bonding structure (Figure 1), due to the debonding of the interface, the fluid (water, air, etc.) enters the debonding gap, forming fluid layer F with a certain thickness. At this time, the debonding interface is a fluid–solid interface, and the bonding structure becomes a “four-layer structure”. Here, the interface between substrate layer A and fluid layer F is marked as interface $I_{2}^{\prime }$, and the interface between adhesive layer B and fluid layer F is marked as interface $I_{2}^{\prime \prime }$, where the thickness and density of the fluid layer are *d*_F_ (m) and *ρ*_F_ (kg/m^3^), respectively.

For fluid layer F, the wave propagation characteristics are similar to those in the liquid domain with *x*_3_ ≤ 0. There is a group of “upward” and “downward” longitudinal waves, and the expressions of displacement and stress are the same as above. We assume that *B*_0_ and *B*_R_ are the amplitudes of the upward and downward longitudinal waves, respectively. For simplicity, it is assumed that fluid layer F is liquid and has the same physical parameters as the liquid domain with *x*_3_ ≤ 0.

When the upper interface of the adhesive layer is debonded and the lower interface is well bonded, interfaces $I_{2}^{\prime }$ and $I_{2}^{\prime \prime }$ are liquid–solid interfaces, and the interface conditions are

(16) $${u_{3}^{A}=u_{3}^{F},\;\sigma_{33}^{A}=\sigma_{33}^{F},\sigma_{13}^{A}=\sigma_{13}^{F}=0,\;\sigma_{23}^{A}=\sigma_{23}^{F}=0|}_{x_{3}=h_{2}^{\prime }}$$

(17) $${u_{3}^{B}=u_{3}^{F},\;\sigma_{33}^{B}=\sigma_{33}^{F},\sigma_{13}^{B}=\sigma_{13}^{F}=0,\;\sigma_{23}^{B}=\sigma_{23}^{F}=0|}_{x_{3}=h_{2}^{\prime \prime }}$$

Simultaneously combining Equations (11), (12), (14), (16), and (17), the global matrix equation is obtained as shown in Equation (18). Among them, block matrices [*P*(0)], [*J*(*x*_3_)] and [*S*(*h*_4_)] are shown in Appendix C.

(18) $$\begin{array}{l} {\left[\begin{array}{cccc} \left[Q^{A}\left(0\right)\right] & & & \left[P(0)\right]\\ \left[Q^{A}\left(h_{2}^{\prime }\right)\right] & & & \left[J(h_{2}^{\prime })\right]\\ & \left[Q^{B}\left(h_{2}^{\prime \prime }\right)\right] & & \left[J(h_{2}^{\prime \prime })\right]\\ & -\left[R_{m}^{B}\left(h_{3}\right)\right] & \left[R_{m}^{C}\left(h_{3}\right)\right] & \\ & & \left[Q^{C}\left(h_{4}\right)\right] & \left[S(h_{4})\right] \end{array}\right]}_{\;22\times 22}\cdot {\left[\begin{array}{c} \left\{N_{i}^{A}/A_{0}\right\}\\ \left\{N_{i}^{B}/A_{0}\right\}\\ \left\{N_{i}^{C}/A_{0}\right\}\\ \left\{\begin{array}{l} B_{0}/A_{0}\\ B_{R}/A_{0}\\ A_{R}/A_{0}\\ A_{T}/A_{0} \end{array}\right\} \end{array}\right]}_{\;22\times 1}\\ ={\left[\begin{array}{c} \varphi \\ K_{w}jk\left(1+\varphi^{2}\right)\\ {\left\{0\right\}}_{20\times 1} \end{array}\right]}_{\;22\times 1}\;,\;(i=1,2,3,4,5,6)\; \end{array}$$

## 3. Ultrasonic Simulation Analysis of the FRP Bonding Structure

### 3.1. Frequency Domain Simulation Analysis

#### 3.1.1. Simulation Model

Taking the defects on the upper interface of the adhesive layer in the FRP bonding structure as an example, a 2.5-dimensional frequency domain simulation model of the FRP bonding structure under various interface forms was established in the COMSOL5.5 finite element simulation software, as shown in Figure 2.

Figure 2a is the model of the perfect bonded structure. The upper and lower matrix materials in the model are T300/914 plates with fiber directions of 0° and 45°, respectively. It is a uniaxial carbon fiber-reinforced composite material, which is a transversely isotropic material with a thickness of 5 mm. The fiber direction angle of the composite plate here is the deflection angle relative to the *x*_1_ axis direction, and the T300/914-0 plate is rotated 45° along the *x*_3_ axis in the *x*_1_ − *x*_2_ plane, which is the T300/914-45 plate. The material parameters of the T300/914-0 composite material board and epoxy adhesive are shown in Table 1 (the elastic constant of T300/914-45 can be obtained by coordinate system transformation, and is not described in detail here). The upper and lower surface areas of the bonding structure are all water, the thickness of the water layer is 3 mm, the density of water is 1000 kg/m^3^, the longitudinal wave velocity c_w_ is 1490 m/s, and the transverse width of the entire model is 8 mm.

Figure 2b is the bonding structure model with a slip interface. From Section 1, the boundary conditions at the liquid–solid interface are the same as those at the slip interface. Therefore, when the simulation model is established, the interface slip is simulated by introducing a thin layer of water between the substrate plate and the adhesive layer. In addition, the geometric dimensions and material parameters of each module in this model are consistent with those of the well-bonded model.

Figure 2c is the bonding structure model with a debonding interface. The interface debonding is simulated by introducing a 1 mm thick water layer between the substrate plate and the adhesive layer. At this time, the thickness of the epoxy adhesive is 2 mm. The geometric dimensions and material parameters of the remaining modules are consistent with those of the well-bonded model.

In the simulation model, a perfectly matched layer (PML) is set on the upper and lower regions of the bonding structure to fully absorb the sound waves at the upper and lower boundaries. The floquet periodic boundary is set at the left and right boundaries of the bonding structure and the upper and lower waters to simulate its infinite extension in the lateral direction. The solid mechanics and pressure acoustics modules are selected in the simulation model to realize the extraction of ultrasonic transmission coefficient.

#### 3.1.2. Introduction of the Slip Interface

**Figure 3:**
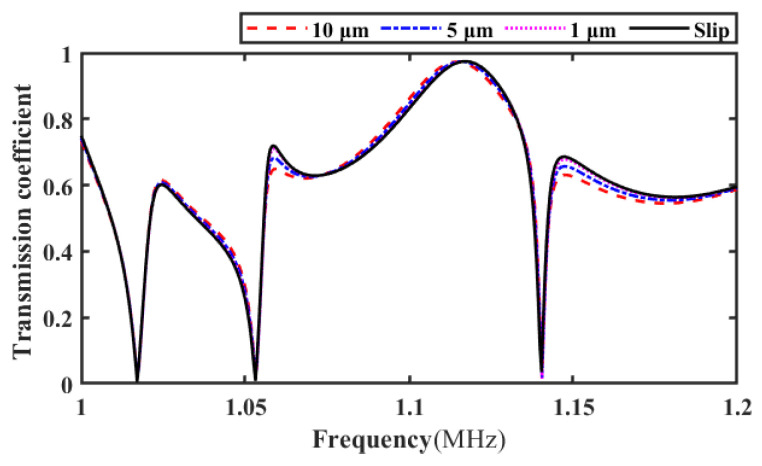
Comparison of transmission coefficient frequency spectrum under water layers of different thicknesses and theoretical calculation results.

In Figure 2b, the interfacial slip is simulated by introducing a thin layer of water between the substrate plate and the adhesive layer. In order to determine the thickness of the thin water layer, water layers with thicknesses of 10 µm, 5 µm, and 1 µm are set here. When the incident angle of the acoustic wave is 20°, the corresponding transmission coefficient frequency spectrum under the three thicknesses is extracted based on the simulation model in Figure 2b and compared with the transmission coefficient spectrum calculated based on the theoretical derivation when the bonding structure interface slips, as shown in Figure 3. In addition, the correlation coefficients between the simulation results and the theoretical calculation results for three different water layer thicknesses are calculated, as shown in Table 2. It can be seen from Table 2 that when the thickness of the liquid thin layer is 10 µm, the correlation coefficient between the simulation and theoretical calculation results reaches 0.9682. This indicates that interfacial slip can be simulated by introducing a water layer with a small thickness (≤10 µm) between the CFRP board and the adhesive layer. Therefore, in order to ensure the accuracy of the simulation results, the thickness of the thin water layer is set to 1 µm when simulating the slip interface.

#### 3.1.3. Transmission Coefficient Spectrum

**Figure 4:**
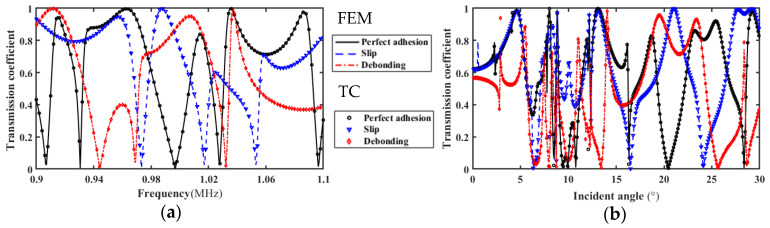
Transmission coefficient frequency spectrum and angle spectrum. (**a**) Transmission coefficient frequency spectrum; (**b**) Angle spectrum.

In order to verify the correctness of theoretical calculation (TC) results, the frequency domain simulation model in Figure 2 is used. When the incident angle of the acoustic wave is 20°, the frequency spectrum of the ultrasonic transmission coefficient when the upper interface of the FRP bonded structure is intact, slipped, and debonding is extracted, and compared with the theoretical calculation results as shown in Figure 4. The frequency spectrum curve calculated by the theory is in good agreement with the frequency spectrum curve calculated by the finite element method (FEM).

### 3.2. Time-Domain Analysis

#### 3.2.1. Simulation Model

**Figure 5:**
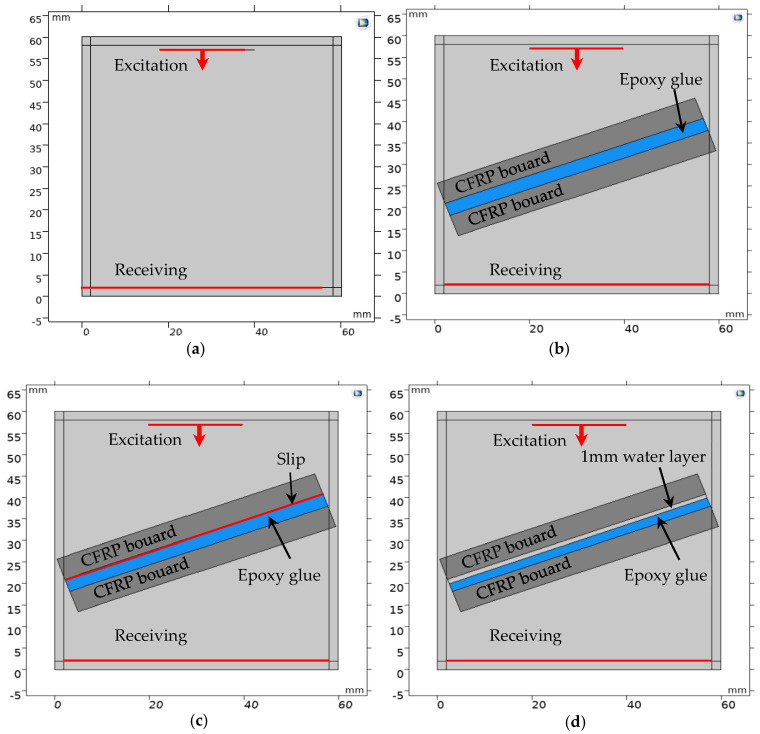
Time−domain finite element simulation model of transmission characteristics. (**a**) Reference signal acquisition model; (**b**) Perfect bonding; (**c**) Interfacial slip; (**d**) Debonding.

The simulation model of acoustic wave excitation and reception without a specimen under water immersion conditions is established, as shown in Figure 5a. The excitation and reception of acoustic wave signals are realized by means of line excitation and line reception. The length of the excitation line is set to 20 mm, the length of the receiving line is 56 mm, the distance between excitation and reception is set to 55 mm, and a perfect matching layer is set around the geometric model to achieve full absorption of sound waves at the model boundary. The pressure acoustics and transient physics modules are selected in the simulation.

On the basis of the pressure acoustics and transient modules in Figure 5a, the solid mechanics and transient modules are added to establish a finite element simulation model of the CFRP bonded structure with intact, slip and debonding interfaces under water immersion conditions, as shown in Figure 5b–d. Among them, the incident angle of sound waves is controlled by rotating the bonding structure. The base layer is selected from the T300/914 material in Table 1, the thickness is 5 mm, and the fiber direction of the uniaxial CFRP board (T300/914) is set to be parallel to the length direction of the board. The adhesive layer selects the epoxy glue in Table 1. In addition, the material thickness of each layer in the bonding structure under the three interface forms is consistent with that in Figure 2. A sine wave with a center frequency of 1 MHz (arbitrary frequency) and Gaussian window modulation is selected as the excitation signal. The finite element model is meshed with free triangles, and the maximum mesh element size is guaranteed to be one-tenth of the wavelength of the acoustic wave propagating in the medium.

#### 3.2.2. Transmission Wave and Transmission Coefficient

**Figure 6:**
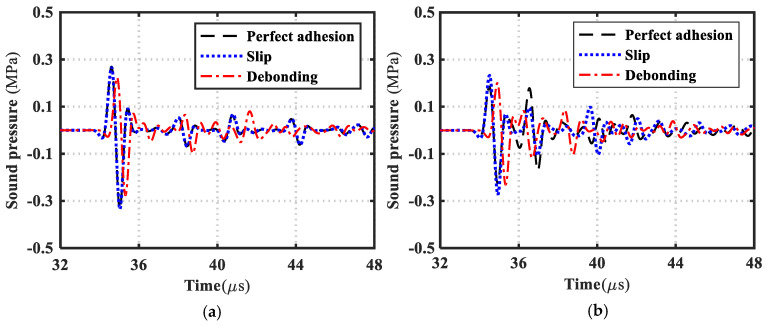
Transmitted waves for good bonding, slippage, and debonding. (**a**) Incidence angle 0°; (**b**) Incidence angle 30°.

Based on the simulation model in Figure 5, when the incident angle of the acoustic wave is 0° and 30°, the corresponding transmitted waves of the CFRP bonded structure when the interface is the rigid connection, slippage, and debonding are extracted and compared, as shown in Figure 6. When the incident angle of the acoustic wave is 0°, the transmission waveform of the well-bonded and the interface slipped transmission waveform is completely coincident, and the transmission waveform of interface debonding is quite different. At this time, when the longitudinal wave is vertically incident, the waveform conversion cannot occur, and there are only longitudinal waves and no transverse waves in the bonding structure. When the incident angle is 30°, the corresponding transmitted waveforms of the three are quite different. To sum up, when the acoustic wave is vertically incident, the slip and debonding interfaces cannot be distinguished by the transmitted wave, and with the increase in the incident angle, the difference between the waveform of the well-bonded transmitted wave and that of the slip is more obvious.

Taking the signal of the receiving end when there is a test piece as the transmitted wave time-domain signal and taking the signal of the direct wave when there is no test piece as the reference time-domain signal, the Fast Fourier transform is performed on them, respectively, to obtain transmittance frequency domain signal *T*(*f*) and reference frequency domain signal *D*(*f*). Transmission coefficient *T* is the ratio of the transmission spectrum signal to the reference frequency domain signal, as shown in Equation (19).

(19) $$T=\frac{T^{\prime }\left(f\right)}{D\left(f\right)}$$

**Figure 7:**
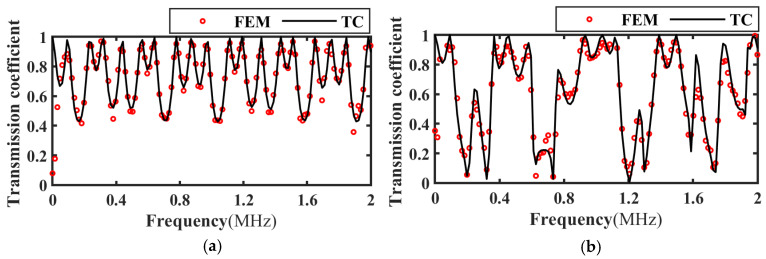
Frequency spectrum of transmission coefficients for perfect bonding at different incidence angles. (**a**) Incidence angle 0°; (**b**) Incidence angle 30°.

**Figure 8:**
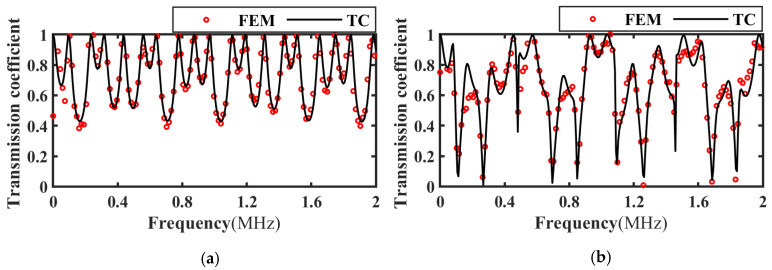
Frequency spectrum of transmission coefficients at different incident angles for slipping specimens. (**a**) Incidence angle 0°; (**b**) Incidence angle 30°.

**Figure 9:**
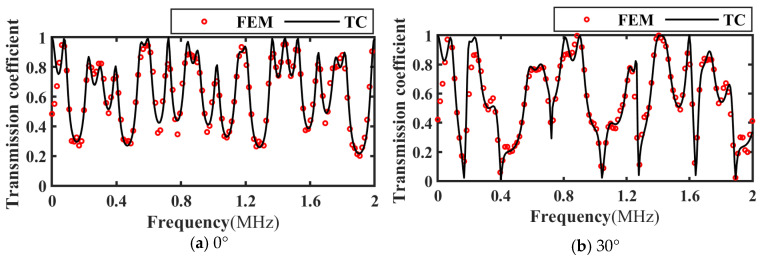
Frequency spectrum of transmission coefficients at different incident angles for debonding specimens. (**a**) Incidence angle 0°; (**b**) Incidence angle 30°.

Based on the transmitted wave time-domain signal in Figure 6, the frequency spectrum of the transmission coefficient of the intact, slipped, and debonded CFRP-bonded structures was calculated using Equation (19) and compared with the theoretical calculation results, as shown in Figure 7, Figure 8 and Figure 9. It can be seen from the figure that when the incident angle is 0° and 30°, the calculated results of the two methods are in good agreement. In addition, when the sound wave is vertically incident, the transmission coefficient spectra corresponding to intact bonding and slipping are resumable; when the sound wave is incident obliquely, the transmission coefficient spectra corresponding to intact bonding, slipping, and debonding are quite different.

## 4. Experimental Measurement and Result Analysis

### 4.1. Preparation of FRP Bonding Specimen

For the CFRP bonding structure with interfacial defects (slip, debonding) to be prepared, the substrate material is the uniaxial CFRP plate (T700/Epoxy). Its geometric size is 120 × 85 × 5 mm^3^, and the carbon fiber direction is parallel to its length direction, and the thickness of the CFRP plate is 5 mm. In order to control the thickness of the adhesive layer, a batch of rectangular frames with thicknesses of 1 mm, 2 mm, and 3 mm are produced by means of 3D printing technology, namely the adhesive layer thickness control mold, and the material type is photosensitive resin.

**Figure 10:**
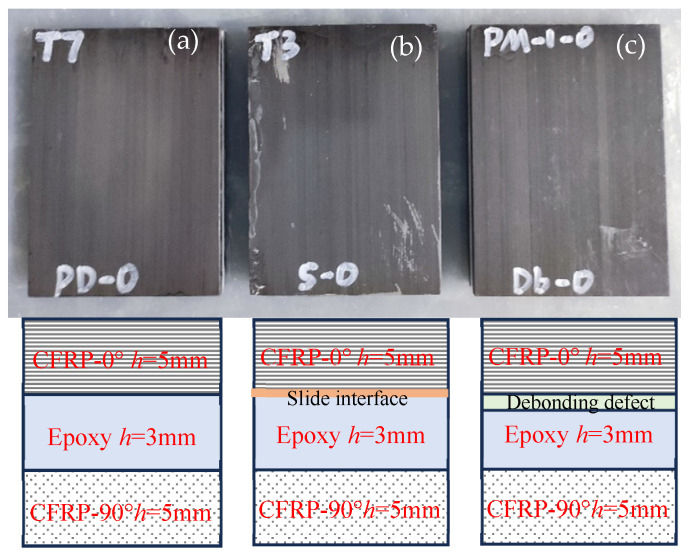
Manufactured CFRP-bonded specimens. (**a**) Perfect bonding; (**b**) Slipping specimens; (**c**) Debonding specimens.

When making a well-bonded specimen, according to the production process of reference [14,15], the surface of the CFRP is first polished with 600-grit sandpaper. The process is initiated by utilizing acetone to degrease the surface of the polished CFRP, and then glue is applied and molded after the surface is dried. According to the operating instructions of the CX2002 epoxy resin adhesive, the bonding specimen is placed at room temperature for 24 h to achieve complete curing of the epoxy resin. When making a bonded test piece with debonding defects, the thickness of the adhesive layer is set to 2 mm and the debonding water layer is set to 1 mm. Upon completion of the operation process with the meticulously prepared bonded test piece, the two substrate plates are secured using clips. When making the adhesive test piece with slip defects, the thickness of the adhesive layer is set to 3 mm, and the same method is adopted as that of making the adhesive test piece with debonding. The prepared bond structure test piece is shown in Figure 10.

In order to measure the viscoelastic properties of uniaxial CFRP panels and epoxy resin adhesives, the phase velocity distributions in the isotropic and anisotropic planes of uniaxial CFRP panels are measured by the water immersion ultrasonic transmission method. Based on this, the real part of the complex elastic constant is obtained. Using the spectral measurement results of the ultrasonic transmission coefficient and selecting the particle swarm optimization algorithm based on simulated annealing, the simultaneous inversion of the real and imaginary parts of the complex elastic constants in the CFRP plate is achieved. Using a similar method, the complex elastic constant of the epoxy block is inverted. The densities of the CFRP plate and the epoxy resin are 1530 kg/m^3^ and 1141 kg/m^3^, respectively, and the corresponding elastic constants are shown in Table 3 and Table 4, respectively.

### 4.2. Experimental Measurement of Ultrasonic Transmission Coefficient Spectrum

**Figure 11:**
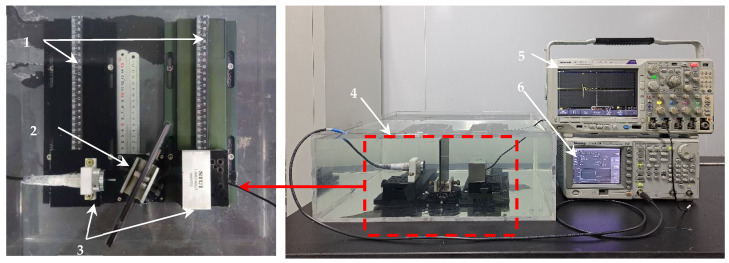
Water immersion ultrasonic transmission wave experimental detection system. 1: Guide; 2: Turntable; 3: Water immersion ultrasonic sensor; 4: Sink; 5: Oscilloscope; 6: Signal generator.

A water immersion transmission wave experimental detection system was built, as shown in Figure 11, which was used to measure the transmission signal and the coefficient spectrum of the CFRP-bonded specimen with interface defects. The system mainly included a function/signal generator, a digital oscilloscope, a water tank, a linear guide rail, and a water immersion ultrasonic sensor. Among them, the model of the excitation sensor was C302 (Olympus, Tokyo, Japan), the diameter is 25 mm, the model of the receiving sensor was 1.0M80×40SJT (SIUI, Shantou Ultrasound, Shantou, China), the size of the end face was 80 × 40 mm^2^, the center frequency of the excitation and receiving sensors was 1 MHz, and the distance between the two was 130 mm. In addition, all experiments in this paper were carried out under normal temperature and pressure, the wave speed c_w_ in water was 1490 m/s and the density was 1000 kg/m^3^.

**Figure 12:**
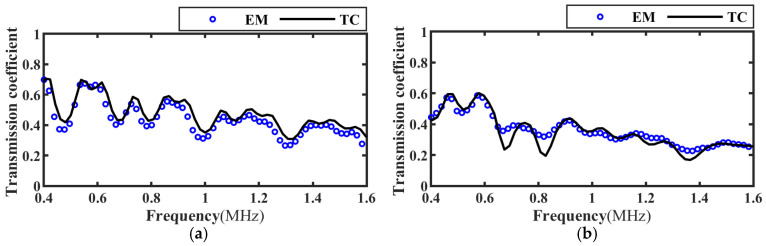
Frequency spectrum of ultrasonic transmission coefficient at perfect bonding. (**a**) Incidence angle 0°; (**b**) Incidence angle 20°.

**Figure 13:**
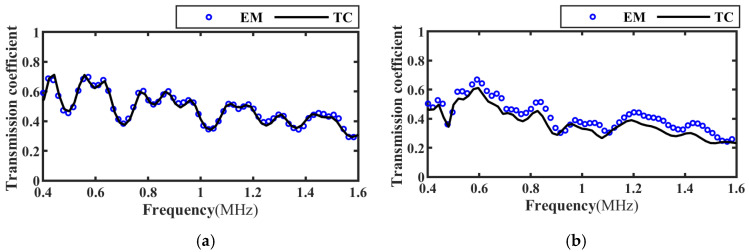
Frequency spectrum of ultrasonic transmission coefficients at interface slip. (**a**) Incidence angle 0°; (**b**) Incidence angle 20°.

**Figure 14:**
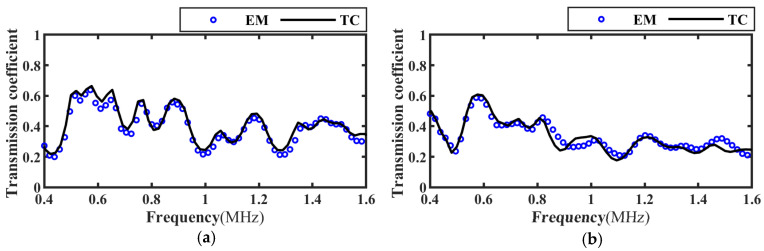
Frequency spectrum of ultrasonic transmission coefficients at debonding specimens. (**a**) Incidence angle 0°; (**b**) Incidence angle 20°.

The transmission wave corresponding to the perfect bonding, the interface on the adhesive layer in the state of slipping and the debonding interface were obtained by experimental measurement (EM), and the corresponding transmission coefficient frequency spectrum can be obtained according to Formula (7). The parameters of the CFRP board and the epoxy adhesive in Table 3 and Table 4 were brought into the simulation model, and the ultrasonic transmission coefficient was extracted and compared with the experimental results, as shown in Figure 12, Figure 13 and Figure 14. Considering the −6 dB bandwidth of the sensor (0.4 MHz~1.6 MHz), the two results were in good agreement.

## 5. Discussion

This study presents a comprehensive analysis of the impact of various bonding interface states on the ultrasonic transmission coefficient spectra of Fiber-Reinforced Polymer (FRP)-bonded structures. Both theoretical and simulation methodologies are utilized. The validity of the theoretical calculation approach is corroborated through frequency-domain simulations, which subsequently guide experimental investigations via time-domain simulations. By integrating experimental measurements with theoretically computed transmission coefficient spectra, it becomes possible to discern the nature of debonding at the bonded interface. The findings substantiate the viability of employing transmission coefficient spectra as a metric for assessing bonding quality.

In our current research work, the use of ultrasonic reflection/transmission coefficients enables the measurement of weak bonding, interfacial slip, and debonding defects in FRP-bonded structures. Looking ahead, rapid identification of debonding defects through the experimental measurement of transmission coefficient spectra, underpinned by theoretical computations and supplemented with intelligent optimization algorithms or neural networks, is envisaged.

## 6. Conclusions

In this paper, an acoustic inspection method based on the ultrasonic transmission coefficient spectrum is developed for the nondestructive testing of adhesive failures in FRP-bonded structures, and the derivation of ultrasonic transmission coefficient expressions, ultrasonic transmission characterization, finite element simulation, and experimental testing are investigated, and the following main conclusions are obtained:

For the ultrasonic propagation problem in composite bonded structures, the analytical model of ultrasonic transmission coefficients in FRP-bonded structures with thick adhesive layers under water immersion conditions when the interface is rigidly connected, slipping and debonding is established based on the global matrix method. The effects of changes in the angle and frequency of acoustic wave incidence on the ultrasonic transmission characteristics are revealed.

In order to verify the correctness of the theoretical calculations and provide guidance for carrying out experimental tests, time-domain and frequency-domain simulation models of ultrasonic transmission from water immersion were established based on the finite element method. The propagation characteristics of the acoustic wave in the bonded structure and the extraction method of ultrasonic transmission characteristics in the time/frequency domain are clarified through simulation analysis.

Specimens exhibiting a variety of bonding failures are meticulously prepared. The ultrasonic transmission coefficients are then measured spectrally using a water-immersion ultrasonic testing system. This approach enables the clear identification and differentiation of bonding defects. The integration of experimental measurements with theoretical calculations further enhances the precision and reliability of these defect characterizations.

## Figures and Tables

**Table 1:** Material properties of T300/914-0 and epoxy resin.

Material	Density/(kg/m^3^)	C_11_/GPa	C_12_/GPa	C_23_/GPa	C_44_/GPa	C_22_/GPa	C_55_/GPa
T300/914-0	1560	143.8	6.2	6.5	3.6	13.3	5.7
Epoxy resin	1170	7.97	5.14	5.14	1.42	7.97	1.42

**Table 2:** Correlation coefficient between simulation calculation and theoretical calculation results.

Water Layer Thickness/µm	Correlation Coefficient
10	0.9682
5	0.9775
1	0.9912

**Table 3:** The elastic constant of the CFRP plate obtained by experimental measurement.

$C_{11}^{*}/\mathrm{GPa}$	$C_{13}^{*}/\mathrm{Gpa}$	$C_{22}^{*}/\mathrm{Gpa}$	$C_{23}^{*}/\mathrm{Gpa}$	$C_{33}^{*}/\mathrm{Gpa}$	$C_{44}^{*}/\mathrm{Gpa}$	$C_{55}^{*}/\mathrm{Gpa}$
131.47 + 8.13i	6.37 + 0.61i	12.84 + 0.47i	6.73 + 0.27i	12.82 + 0.36i	3.09 + 0.15i	5.04 + 0.54i

**Table 4:** The elastic constants of epoxy resin adhesives obtained by experimental measurement.

$C_{11}^{*}/\mathrm{GPa}$	$C_{13}^{*}/\mathrm{Gpa}$	$C_{22}^{*}/\mathrm{Gpa}$	$C_{23}^{*}/\mathrm{Gpa}$	$C_{33}^{*}/\mathrm{Gpa}$	$C_{44}^{*}/\mathrm{Gpa}$ $C_{44}^{*}/\mathrm{GPa}$	$C_{55}^{*}/\mathrm{Gpa}$
7.77 + 0.26i	4.69 + 0.17i	7.77 + 0.26i	4.69 + 0.17i	7.77 + 0.26i	1.55 + 0.12i	1.55 + 0.12i

## Data Availability

The data presented in this study are available on request from the corresponding author.

## Appendix A

$$\left\{ \begin{array}{l} R_{11}=W_{1}^{1}\cdot e^{jk(x_{1}+\alpha_{1}x_{3}-ct)}\\ R_{12}=W_{1}^{2}\cdot e^{jk(x_{1}+\alpha_{2}x_{3}-ct)}\\ R_{13}=W_{1}^{3}\cdot e^{jk(x_{1}+\alpha_{3}x_{3}-ct)}\\ R_{14}=W_{1}^{4}\cdot e^{jk(x_{1}+\alpha_{4}x_{3}-ct)}\\ R_{15}=W_{1}^{5}\cdot e^{jk(x_{1}+\alpha_{5}x_{3}-ct)}\\ R_{16}=W_{1}^{6}\cdot e^{jk(x_{1}+\alpha_{6}x_{3}-ct)} \end{array}\right.,\left\{ \begin{array}{l} R_{21}=W_{2}^{1}\cdot e^{jk(x_{1}+\alpha_{1}x_{3}-ct)}\\ R_{22}=W_{2}^{2}\cdot e^{jk(x_{1}+\alpha_{2}x_{3}-ct)}\\ R_{23}=W_{2}^{3}\cdot e^{jk(x_{1}+\alpha_{3}x_{3}-ct)}\\ R_{24}=W_{2}^{4}\cdot e^{jk(x_{1}+\alpha_{4}x_{3}-ct)}\\ R_{25}=W_{2}^{5}\cdot e^{jk(x_{1}+\alpha_{5}x_{3}-ct)}\\ R_{26}=W_{2}^{6}\cdot e^{jk(x_{1}+\alpha_{6}x_{3}-ct)} \end{array}\right.,\left\{ \begin{array}{l} R_{31}=W_{3}^{1}\cdot e^{jk(x_{1}+\alpha_{1}x_{3}-ct)}\\ R_{32}=W_{3}^{2}\cdot e^{jk(x_{1}+\alpha_{2}x_{3}-ct)}\\ R_{33}=W_{3}^{3}\cdot e^{jk(x_{1}+\alpha_{3}x_{3}-ct)}\\ R_{34}=W_{3}^{4}\cdot e^{jk(x_{1}+\alpha_{4}x_{3}-ct)}\\ R_{35}=W_{3}^{5}\cdot e^{jk(x_{1}+\alpha_{5}x_{3}-ct)}\\ R_{36}=W_{3}^{6}\cdot e^{jk(x_{1}+\alpha_{6}x_{3}-ct)} \end{array}\right.$$

$$\left\{ \begin{array}{l} R_{41}=jk\cdot e^{jk(x_{1}+\alpha_{1}x_{3}-ct)}\left(C_{31}\cdot W_{1}^{1}+C_{33}\cdot W_{3}^{1}\alpha_{1}+C_{34}\cdot W_{2}^{1}\alpha_{1}+C_{35}\cdot W_{1}^{1}\alpha_{1}+C_{35}\cdot W_{3}^{1}+C_{36}\cdot W_{2}^{1}\right)\\ R_{42}=jk\cdot e^{jk(x_{1}+\alpha_{2}x_{3}-ct)}\left(C_{31}\cdot W_{1}^{2}+C_{33}\cdot W_{3}^{2}\alpha_{2}+C_{34}\cdot W_{2}^{2}\alpha_{2}+C_{35}\cdot W_{1}^{2}\alpha_{2}+C_{35}\cdot W_{3}^{2}+C_{36}\cdot W_{2}^{2}\right)\\ R_{43}=jk\cdot e^{jk(x_{1}+\alpha_{3}x_{3}-ct)}\left(C_{31}\cdot W_{1}^{3}+C_{33}\cdot W_{3}^{3}\alpha_{3}+C_{34}\cdot W_{2}^{3}\alpha_{3}+C_{35}\cdot W_{1}^{3}\alpha_{3}+C_{35}\cdot W_{3}^{3}+C_{36}\cdot W_{2}^{3}\right)\\ R_{44}=jk\cdot e^{jk(x_{1}+\alpha_{4}x_{3}-ct)}\left(C_{31}\cdot W_{1}^{4}+C_{33}\cdot W_{3}^{4}\alpha_{4}+C_{34}\cdot W_{2}^{4}\alpha_{4}+C_{35}\cdot W_{1}^{4}\alpha_{4}+C_{35}\cdot W_{3}^{4}+C_{36}\cdot W_{2}^{4}\right)\\ R_{45}=jk\cdot e^{jk(x_{1}+\alpha_{5}x_{3}-ct)}\left(C_{31}\cdot W_{1}^{5}+C_{33}\cdot W_{3}^{5}\alpha_{5}+C_{34}\cdot W_{2}^{5}\alpha_{5}+C_{35}\cdot W_{1}^{5}\alpha_{5}+C_{35}\cdot W_{3}^{5}+C_{36}\cdot W_{2}^{5}\right)\\ R_{46}=jk\cdot e^{jk(x_{1}+\alpha_{6}x_{3}-ct)}\left(C_{31}\cdot W_{1}^{6}+C_{33}\cdot W_{3}^{6}\alpha_{6}+C_{34}\cdot W_{2}^{6}\alpha_{6}+C_{35}\cdot W_{1}^{6}\alpha_{6}+C_{35}\cdot W_{3}^{6}+C_{36}\cdot W_{2}^{6}\right) \end{array}\right.$$

$$\left\{ \begin{array}{l} R_{51}=jk\cdot e^{jk(x_{1}+\alpha_{1}x_{3}-ct)}\left(C_{51}\cdot W_{1}^{1}+C_{53}\cdot W_{3}^{1}\alpha_{1}+C_{54}\cdot W_{2}^{1}\alpha_{1}+C_{55}\cdot W_{1}^{1}\alpha_{1}+C_{55}\cdot W_{3}^{1}+C_{56}\cdot W_{2}^{1}\right)\\ R_{52}=jk\cdot e^{jk(x_{1}+\alpha_{2}x_{3}-ct)}\left(C_{51}\cdot W_{1}^{2}+C_{53}\cdot W_{3}^{2}\alpha_{2}+C_{54}\cdot W_{2}^{2}\alpha_{2}+C_{55}\cdot W_{1}^{2}\alpha_{2}+C_{55}\cdot W_{3}^{2}+C_{56}\cdot W_{2}^{2}\right)\\ R_{53}=jk\cdot e^{jk(x_{1}+\alpha_{3}x_{3}-ct)}\left(C_{51}\cdot W_{1}^{3}+C_{53}\cdot W_{3}^{3}\alpha_{3}+C_{54}\cdot W_{2}^{3}\alpha_{3}+C_{55}\cdot W_{1}^{3}\alpha_{3}+C_{55}\cdot W_{3}^{3}+C_{56}\cdot W_{2}^{3}\right)\\ R_{54}=jk\cdot e^{jk(x_{1}+\alpha_{4}x_{3}-ct)}\left(C_{51}\cdot W_{1}^{4}+C_{53}\cdot W_{3}^{4}\alpha_{4}+C_{54}\cdot W_{2}^{4}\alpha_{4}+C_{55}\cdot W_{1}^{4}\alpha_{4}+C_{55}\cdot W_{3}^{4}+C_{56}\cdot W_{2}^{4}\right)\\ R_{55}=jk\cdot e^{jk(x_{1}+\alpha_{5}x_{3}-ct)}\left(C_{51}\cdot W_{1}^{5}+C_{53}\cdot W_{3}^{5}\alpha_{5}+C_{54}\cdot W_{2}^{5}\alpha_{5}+C_{55}\cdot W_{1}^{5}\alpha_{5}+C_{55}\cdot W_{3}^{5}+C_{56}\cdot W_{2}^{5}\right)\\ R_{56}=jk\cdot e^{jk(x_{1}+\alpha_{6}x_{3}-ct)}\left(C_{51}\cdot W_{1}^{6}+C_{53}\cdot W_{3}^{6}\alpha_{6}+C_{54}\cdot W_{2}^{6}\alpha_{6}+C_{55}\cdot W_{1}^{6}\alpha_{6}+C_{55}\cdot W_{3}^{6}+C_{56}\cdot W_{2}^{6}\right) \end{array}\right.$$

$$\left\{ \begin{array}{l} R_{61}=jk\cdot e^{jk(x_{1}+\alpha_{1}x_{3}-ct)}\left(C_{41}\cdot W_{1}^{1}+C_{43}\cdot W_{3}^{1}\alpha_{1}+C_{44}\cdot W_{2}^{1}\alpha_{1}+C_{45}\cdot W_{1}^{1}\alpha_{1}+C_{45}\cdot W_{3}^{1}+C_{46}\cdot W_{2}^{1}\right)\\ R_{62}=jk\cdot e^{jk(x_{1}+\alpha_{2}x_{3}-ct)}\left(C_{41}\cdot W_{1}^{2}+C_{43}\cdot W_{3}^{2}\alpha_{2}+C_{44}\cdot W_{2}^{2}\alpha_{2}+C_{45}\cdot W_{1}^{2}\alpha_{2}+C_{45}\cdot W_{3}^{2}+C_{46}\cdot W_{2}^{2}\right)\\ R_{63}=jk\cdot e^{jk(x_{1}+\alpha_{3}x_{3}-ct)}\left(C_{41}\cdot W_{1}^{3}+C_{43}\cdot W_{3}^{3}\alpha_{3}+C_{44}\cdot W_{2}^{3}\alpha_{3}+C_{45}\cdot W_{1}^{3}\alpha_{3}+C_{45}\cdot W_{3}^{3}+C_{46}\cdot W_{2}^{3}\right)\\ R_{64}=jk\cdot e^{jk(x_{1}+\alpha_{4}x_{3}-ct)}\left(C_{41}\cdot W_{1}^{4}+C_{43}\cdot W_{3}^{4}\alpha_{4}+C_{44}\cdot W_{2}^{4}\alpha_{4}+C_{45}\cdot W_{1}^{4}\alpha_{4}+C_{45}\cdot W_{3}^{4}+C_{46}\cdot W_{2}^{4}\right)\\ R_{65}=jk\cdot e^{jk(x_{1}+\alpha_{5}x_{3}-ct)}\left(C_{41}\cdot W_{1}^{5}+C_{43}\cdot W_{3}^{5}\alpha_{5}+C_{44}\cdot W_{2}^{5}\alpha_{5}+C_{45}\cdot W_{1}^{5}\alpha_{5}+C_{45}\cdot W_{3}^{5}+C_{46}\cdot W_{2}^{5}\right)\\ R_{66}=jk\cdot e^{jk(x_{1}+\alpha_{6}x_{3}-ct)}\left(C_{41}\cdot W_{1}^{6}+C_{43}\cdot W_{3}^{6}\alpha_{6}+C_{44}\cdot W_{2}^{6}\alpha_{6}+C_{45}\cdot W_{1}^{6}\alpha_{6}+C_{45}\cdot W_{3}^{6}+C_{46}\cdot W_{2}^{6}\right) \end{array}\right.$$

## Appendix B

Partitioned matrix:

$$\left[G(0)\right]=\left[\begin{array}{cc} \varphi & 0\\ -K_{w}jk\left(1+\varphi^{2}\right) & 0\\ 0 & 0\\ 0 & 0 \end{array}\right]\left[H(h_{4})\right]=\left[\begin{array}{cc} 0 & -\varphi \cdot e^{jk\left(\varphi \cdot h_{4}\right)}\\ 0 & -K_{w}jk\left(1+\varphi^{2}\right)\cdot e^{jk\left(\varphi \cdot h_{4}\right)}\\ 0 & 0\\ 0 & 0 \end{array}\right]$$

Matrices [*L*^A^(*h*_2_)] and [*K*^B^(*h*_2_)] are 6 × 6 matrices corresponding to base layer A and adhesive layer B, respectively, and some elements of the matrix are taken from the third to sixth rows of material property matrix [*R*(*x*_3_)] in Appendix B.

$$L^{A}(h_{2})=\left[\begin{array}{cccccc} R_{31}^{A} & R_{32}^{A} & R_{33}^{A} & R_{34}^{A} & R_{35}^{A} & R_{36}^{A}\\ R_{41}^{A} & R_{42}^{A} & R_{43}^{A} & R_{44}^{A} & R_{45}^{A} & R_{46}^{A}\\ 0 & 0 & 0 & 0 & 0 & 0\\ 0 & 0 & 0 & 0 & 0 & 0\\ R_{51}^{A} & R_{52}^{A} & R_{53}^{A} & R_{54}^{A} & R_{55}^{A} & R_{56}^{A}\\ R_{61}^{A} & R_{62}^{A} & R_{63}^{A} & R_{64}^{A} & R_{65}^{A} & R_{66}^{A} \end{array}\right]|\begin{array}{c} x_{3}=h_{2} \end{array}$$

$$K^{B}(h_{2})=\left[\begin{array}{cccccc} R_{31}^{B} & R_{32}^{B} & R_{33}^{B} & R_{34}^{B} & R_{35}^{B} & R_{36}^{B}\\ R_{41}^{B} & R_{42}^{B} & R_{43}^{B} & R_{44}^{B} & R_{45}^{B} & R_{46}^{B}\\ R_{51}^{B} & R_{52}^{B} & R_{53}^{B} & R_{54}^{B} & R_{55}^{B} & R_{56}^{B}\\ R_{61}^{B} & T_{62}^{B} & R_{63}^{B} & R_{64}^{B} & R_{65}^{B} & R_{66}^{B}\\ 0 & 0 & 0 & 0 & 0 & 0\\ 0 & 0 & 0 & 0 & 0 & 0 \end{array}\right]|\begin{array}{c} x_{3}=h_{2} \end{array}$$

## Appendix C

Partitioned matrix:

$$P(0)=\left[\begin{array}{cccc} 0 & 0 & \beta & 0\\ 0 & 0 & -K_{w}jk\left(1+\beta^{2}\right) & 0\\ 0 & 0 & 0 & 0\\ 0 & 0 & 0 & 0 \end{array}\right]$$

$$J(x_{3})=\left[\begin{array}{cccc} -\gamma \cdot e^{jk\gamma x_{3}} & \gamma \cdot e^{-jk\gamma x_{3}} & 0 & 0\\ -K_{F}jk\left(1+\gamma^{2}\right)\cdot e^{jk\gamma x_{3}} & -K_{F}jk\left(1+\gamma^{2}\right)\cdot e^{-jk\gamma x_{3}} & 0 & 0\\ 0 & 0 & 0 & 0\\ 0 & 0 & 0 & 0 \end{array}\right]$$

$$S(h_{4})=\left[\begin{array}{cccc} 0 & 0 & 0 & -\varphi \cdot e^{jk(\varphi \cdot h_{4})}\\ 0 & 0 & 0 & -K_{w}jk\left(1+\varphi^{2}\right)\cdot e^{jk(\varphi \cdot h_{4})}\\ 0 & 0 & 0 & 0\\ 0 & 0 & 0 & 0 \end{array}\right]$$
